# Feline Immunodeficiency Virus Neuropathogenesis: A Model for HIV-Induced CNS Inflammation and Neurodegeneration

**DOI:** 10.3390/vetsci4010014

**Published:** 2017-03-06

**Authors:** Rick B. Meeker, Lola Hudson

**Affiliations:** 1 Department of Neurology, University of North Carolina, Chapel Hill, NC 27599, USA; 2 Department of Molecular Biomedical Sciences, College of Veterinary Medicine, North Carolina State University, Raleigh, NC 27607, USA; lola_hudson@ncsu.edu

**Keywords:** AIDS, human immunodeficiency virus, dementia, neurons, microglia, macrophages

## Abstract

Feline Immunodeficiency virus (FIV), similar to its human analog human immunodeficiency virus (HIV), enters the central nervous system (CNS) soon after infection and establishes a protected viral reservoir. The ensuing inflammation and damage give rise to varying degrees of cognitive decline collectively known as HIV-associated neurocognitive disorders (HAND). Because of the similarities to HIV infection and disease, FIV has provided a useful model for both in vitro and in vivo studies of CNS infection, inflammation and pathology. This mini review summarizes insights gained from studies of early infection, immune cell trafficking, inflammation and the mechanisms of neuropathogenesis. Advances in our understanding of these processes have contributed to the development of therapeutic interventions designed to protect neurons and regulate inflammatory activity.

## 1. Introduction

FIV, similar to HIV, is a lentivirus that infects and replicates in T cells and cells of monocyte lineage including perivascular macrophages and microglia [1,2,3,4,5,6,7,8,9,10,11]. Soon after initial systemic infection with HIV or FIV, the virus enters the central nervous system (CNS). During infection, the virus inserts its RNA core into the host cell, which is then reverse transcribed into DNA and integrated into the host cell genome. Since both T cell and monocytoid cells can be long lived, they can maintain a virus reservoir within infected tissues including the CNS. Antiretroviral therapy targeted to various steps in the virus lifecycle can now suppress systemic viral burden to low or undetectable levels and has greatly increased survival in infected individuals. However, because antiretroviral drugs penetrate the blood-brain barrier poorly and are substrates for active efflux transporters, the virus persists in the brain and has led to an increasing prevalence of cognitive dysfunction as the infected population ages [12,13,14,15], even with suppression of both the systemic and cerebrospinal fluid (CSF) viral load [16]. Substantial effort is focused on the development of strategies that will eliminate virus from cellular reservoirs. In the CNS the self-sustaining nature of the microglial population [17,18] and the ability of monocytic cells to sustain viral replication in the absence of T cells [19] make eradication of virus a daunting task. To reduce or eliminate CNS effects of lentiviral infection, we need a better understanding of viral replication in the CNS and the mechanisms that underlie neuronal damage. Because of the difficulty of exploring these processes in humans, animal models have played an essential role in this endeavor.

## 2. FIV Infection of the Nervous System

### 2.1. General Background

FIV was isolated from naturally infected domestic cats soon after the identification of HIV [20] and was subsequently shown to be genetically and functionally similar to HIV including similar cell tropism and the ability to produce a severe acquired immune deficiency syndrome (AIDS) [21]. Many investigators have explored the virology, immunology and pathogenesis of FIV to gain insights into treatment strategies for both veterinary and human medicine. Among the many achievements, these efforts led to approval of an effective FIV vaccine in 2002. Recent studies have addressed important issues such as mechanisms of FIV transmission, replication and evolution [22,23,24,25,26,27], FIV vaccine efficacy [28,29], the role of neutralizing antibodies in disease progression [28,30,31], virus assembly and release [32,33], viral latency and eradication strategies [34,35,36], development of adjunctive therapies [37,38,39,40], immunology [41,42,43,44,45], interactions with the microbiome [46,47], development of antiretroviral compounds [48,49,50] and development of protocols for assessing feline cognitive function [51,52,53,54]. Many of these efforts are summarized in this volume and in a review by Bienzle [55]. In the following sections, we summarize in vitro and in vivo studies of FIV that have provided insights into infection and pathogenesis in the central nervous system (CNS).

In contrast to HIV-1, FIV uses CD134 as a primary receptor instead of CD4 [56,57]. Similar to HIV-1, FIV uses the alpha chemokine receptor, CXCR4, as a co-receptor [58,59,60,61,62]. Although most primary isolates of FIV will infect both T-lymphocytes and monocytes, productive replication in vivo is predominantly within FIV-infected T-lymphocytes [2,63,64,65].

FIV and HIV are thought to gain initial access to the CNS via trafficking of infected monocytes although other mechanisms may contribute such as trafficking of T cells, penetration of free virus across a damaged blood–brain barrier and trafficking across the blood-cerebrospinal fluid (CSF) barrier. Once the virus gains access to the CNS, infection spreads to microglia and astrocytes but not neurons. Virus production in the CNS is typically low and may be controlled in part by CD8+ T cells [66,67,68,69]. Early studies of FIV tropism demonstrated that virus could be recovered from brain and CSF of infected cats [70,71,72,73]. In vitro studies of CNS cell tropism showed that FIV can infect feline astrocytes [70,74,75,76], microglia [6,70,75], choroid plexus macrophages [77] and perhaps brain microvascular endothelial cells [78]. In vivo studies indicated that most virus production in the CNS is maintained by macrophages and microglia [2,6]. However, although microglia and macrophages are primary targets of FIV in the brain and choroid plexus, they support a relatively low level of productive infection [6,77,79] and show negligible cell death. This slow virus production in viable microglia or macrophages can infect peripheral blood mononuclear cells (PBMCs) with high efficiency, rapidly leading to a robust productive infection [6,77]. This can be a source of peripheral infection when antiretroviral therapy is non-compliant or discontinued even when systemic virus levels are undetectable.

One of the questions of interest has been whether virus in the CNS represents uniquely adapted quasispecies that may account for both neurotropism and neurovirulence. Studies of FIV envelope diversity during the early stages of infection have shown that the virus that first appears in the CSF is similar to plasma [80]. Over time there can be significant divergence in the pattern of variants in the CSF versus plasma including the rare appearance of unique variants. However, similar to humans, the profile of FIV quasispecies in the CSF in long-term infection is largely composed of variants in plasma with minor contributions from unique variants indicating that the contribution from virus production within the CNS is quite low. Brain-specific FIV sequences have been isolated from the CSF [71,81] that exhibit greater neurovirulence but evidence for consistent selection of specific FIV variants within the CNS is lacking. Thus, infection of the feline CNS closely parallels HIV infection of the human CNS but evidence for unique strains that are responsible for CNS dysfunction is limited. How viral infection is sustained in the nervous system has been an important but elusive question. With the discovery that microglia are a self-sustaining population of cells within the brain [17,18] and monocytic cell infection can be maintained in the absence of T cells [19] it is expected that microglia will have the capacity to maintain the HIV/FIV reservoir indefinitely. Exploration of the mechanisms that control CNS viral replication remains an important area of research essential to the development of strategies to eradicate virus and the FIV model provides a versatile system for the investigation of these processes through all stages of pathogenesis.

### 2.2. FIV Trafficking across the Blood-Brain Barrier

In vitro studies using fetal feline brain microvascular endothelial cells grown on transwell insert membranes containing 5 µm pores have been used to investigate trafficking of immune cells across the feline blood-brain barrier [82]. These studies used various combinations of astrocytes and/or microglia on the opposite side of the membrane to evaluate the role of parenchymal cells in the trafficking of PBMCs [83,84]. Monocytes, CD4+ T cells, CD8+ T cells and B cells were all observed to cross the endothelium. The transmigration was facilitated by the presence of astrocytes suggesting that although these cells contribute to the blood-brain barrier they also provide signals that support transmigration. The inflammatory cytokine, tumor necrosis factor alpha (TNFα) may provide one such signal as it promotes the transmigration of virus and both infected and uninfected lymphocytes [84]. Exposure of the endothelial/astrocyte cultures to FIV did not induce any preferential trafficking of monocytes, in agreement with previous trafficking studies of human monocytes infected with HIV-1 [85]. Of all the major PBMC subsets, only CD8+ T cells were subject to positive regulation when the feline cultures were exposed to FIV. This observation was consistent with the role of CD8 T cells in the control of CNS infection [66,67,68,69]. When microglia were added to the endothelial/astrocyte cultures the trafficking of T cells, B cells and monocytes was suppressed [83]. The suppression of monocyte trafficking by the microglia was partially reversed when the cultures were inoculated with FIV indicating a largely unexplored role of microglia in the control of immune cell trafficking. The range of trafficking cells seen in vitro was consistent with in vivo trafficking studies that showed early invasion of T cells and B cells within the brain of cats 8–10 weeks after infection with FIV_GL8_ [86]. FIV provirus was measured in the brain as early as two to four weeks post-inoculation indicating that the early immune cell trafficking carries virus into the CNS [87,88]. FIV replication was confirmed by the presence of FIV RNA at 10 weeks when the infiltration of mononuclear cells was at a peak [88]. However, the tissue viral loads and pathology decreased over time indicating significant recovery from this acute phase of infection. Although the ability of FIV or HIV to establish a latent infection in the CNS is controversial, several studies have reported a sustained proviral burden in FIV-infected cats in the brain [72,89] even in the absence of significant viral RNA.

### 2.3. FIV Trafficking across the Blood–CSF Barrier

In addition to penetration across the blood-brain barrier, virus may also penetrate into the brain via the blood-CSF barrier. This barrier differs from the blood–brain barrier as there are no tight junctions at the vascular endothelium. This allows easier penetration of virus and immune cells into the choroid plexus stroma which contains a large population of macrophages and dendritic cells, excellent targets for infection by FIV. The blood-CSF barrier is formed by tight junctions between the cuboidal epithelial cells of the choroid plexus [82]. Trafficking of choroid plexus macrophages across the epithelial barrier can be quite robust and is positively modulated by FIV [90]. In studies of mouse choroid plexus, the epithelium has been shown to constitutively express intracellular adhesion molecule-1 (ICAM-1) and vascular cell adhesion molecule-1 (VCAM-1) which increase in response to inflammatory stimuli [91]. These molecules support the trafficking of cells into the cerebral ventricles and CSF. In both HIV and FIV infection, a relatively robust infiltration of mononuclear cells was seen in the choroid plexus, meninges and subarachnoid space as early as four weeks post-infection [86,92,93,94]. This influx is consistent with studies of envelope sequence diversity described above which showed that most FIV/HIV in the CSF is likely to be of systemic origin [80,95,96,97].

Interactions between macrophages and T cells could be particularly prominent within perivascular regions and within the choroid plexus where infected macrophages are commonly seen in HIV, simian immunodeficiency virus (SIV) and FIV infections. In addition, many studies have documented an admixture of systemic and CNS lentiviral transcripts within the choroid plexus of infected hosts [98,99,100,101] suggesting the potential interaction of infected perivascular macrophages and trafficking T cells. Thus, the choroid plexus is an important interface between the systemic immune system and the brain but the functional role of the choroid plexus in immune cell trafficking and the fate of trafficking cells are still poorly understood. One study suggests that trafficking through the choroid plexus may confer a unique phenotype during recovery of the nervous system after injury [102]. In unpublished studies we have demonstrated the potency of trafficking macrophages after infusion of cultured, FIV-infected feline choroid plexus macrophages into the lateral ventricle of naïve cats. These experiments were designed to evaluate the role of infected macrophage trafficking across the blood-CSF barrier in the early establishment of CNS infection. Although we saw no evidence for CNS infection via this route a high dose of macrophages (3.6 × 10^5^ macrophages/200 µL into the lateral ventricle) induced a massive influx of neutrophils throughout the brain in the absence of local infusion damage or bleeding. The effect was subsequently confirmed in rats using macrophages tagged with fluorescent polystyrene 1 µm spheres. The macrophages were tracked to the cervical lymph nodes but were not seen in the brain parenchyma. This observation, although limited, is an indication of the profound effect ventricular macrophages can have on immune cell trafficking into the brain. It is also a caution that the ventricular environment is an immunologically active environment. This should be taken into consideration in experiments employing intracranial infusions. Important questions remain concerning the sentinel functions of ventricular macrophages and their specific role in the regulation of the CNS immune environment.

## 3. FIV and Neuropathogenesis

### 3.1. Neurological Signs

Because the course of FIV-induced disease, including CNS infection, parallels HIV infection, FIV has been used as a model to investigate the processes that lead to neural damage. One of the difficulties associated with the use of FIV as a model for HIV-associated neurodegenerative disease is the very gradual decline in CNS function with few overt signs until after about 5–8 years, often overlapping with the development of acquired immune deficiency syndrome (AIDS). In addition, as in HIV-infected humans, only a small subset of infected cats show readily apparent neurological signs as they become immune deficient. A question often raised is whether cats naturally infected with FIV have neurological disease since there is little indication of clinically relevant neurological deficits. The brains of random source FIV infected cats examined post mortem show clear signs of degeneration. Less is known about well maintained domestic cats and several factors complicate the assessment of these cats. The deficits seen in HIV infected patients are cognitive-motor processing deficits with no primary motor deficits. In patients maintained on antiretroviral therapy cognitive decline reflects frontal cortex deficits such as attention and executive functions [14,15]. These deficits would not easily be picked up in routine neurological exams of cats (similar for humans where neuropsychological tests are often required to document deficits). It is also likely that such deficits would impact the lives of cats far less than humans. Behavioral deficits seen in immune deficient cats may also be overlooked in the context of opportunistic infections. As cats age the impact of FIV infection is likely to become greater but this remains to be tested.

**Figure 1:**
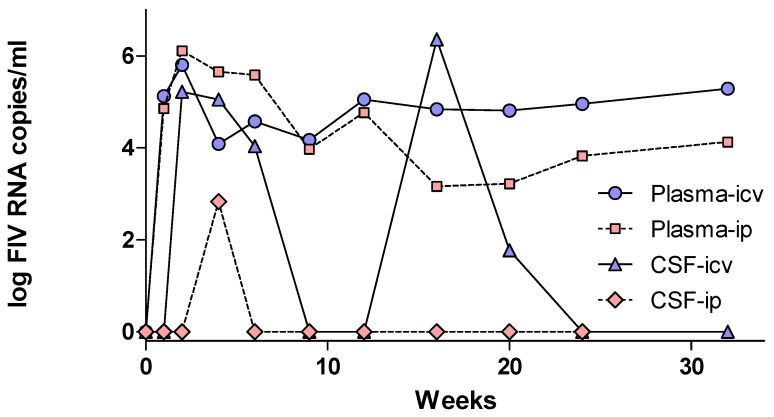
Example of the development of FIV titers in plasma and CSF after systemic (ip) or intracereroventricular (ICV) inoculation of specific pathogen free cats with 2 × $10^{5}$ copies of FIV. In both cases plasma viremia develops rapidly, peaking at 1–2 weeks post-inoculation. The sustained plasma viral load is maintained at higher levels after ICV inoculation (blue circles). CSF viral titers (blue triangles) closely parallel the acute plasma virus and occasionally exceeds plasma titers indicating a CNS origin. Chronic CSF FIV titers may show independent spikes of activity and generally average about $10^{3}$–$10^{4}$ copies/mL. Following systemic inoculation, FIV titers in CSF show a modest delayed peak followed by relative recovery to low levels.

In contrast to the clinical setting, neural and behavioral dysfunction are commonly seen in experimentally infected cats using sensitive physiological and behavioral methods. Because these methods are complex and/or expensive they are not practical in the clinic. In addition, because of the slow course and low natural prevalence of neural deficits, experimental studies of FIV-associated neurological disease have adopted strategies to accelerate neuropathogenesis. These strategies have employed neonatal inoculation with FIV [81], the use of neurovirulent strains of FIV [73,81,103,104,105,106,107] and a combination of neurovirulent strains and direct intracranial inoculation [80]. An example of a typical infection following direct inoculation into the lateral ventricle is illustrated in Figure 1. A rapid and robust FIV production is seen in CSF that meets and often exceeds levels in plasma. Cats inoculated systemically typically show a delayed appearance of virus in CSF with much lower titers, even though the plasma FIV titers are similar to intracranial inoculation. FIV titers in the CSF can vary substantially and the correlation between plasma and CSF virus is relatively low. Spikes of FIV can appear in the CSF in the absence of changes in plasma FIV suggesting independent control of replication.

In studies of experimentally infected specific pathogen free cats, designed to mimic HIV infection in humans, neurological signs can be detected as early as 12 months post infection. The signs include abnormal, stereotypic motor behaviors, anisocoria, increased aggression, increased cortical slow wave activity in quantitative electroencephalograms, prolonged latencies in brainstem evoked potentials, delayed righting and pupillary reflexes, decreased nerve conduction velocities, changes in sleep architecture and deficits in cognitive-motor functions [52,54,104,105,106,107,108,109,110]. Proton magnetic resonance spectroscopy (MRS) demonstrated reductions in the concentrations of the neuronal marker, N-acetyl-aspartate (NAA) and the NAA/choline or NAA/creatine ratio within the brains of FIV-infected cats [81,106]. Neurotoxic activity in the CSF of FIV-infected cats could be detected as early as 4 months after intracranial inoculation [111] prior to any observable deficits. These observations highlight the early and progressive nature of the disease and the importance of early therapeutic intervention to suppress neurodegeneration, particularly in HIV-infected patients. The feline model continues to make important contributions to the development and testing of such interventions. As discussed below, there is an expectation that FIV infection will contribute to the development of age-associated CNS disease including the development of Alzheimer-like pathologies. A valuable contribution to our understanding of the interaction between infection and aging could be made through the development of simple behavioral and/or physiological tests that could routinely be used in veterinary clinics. Information on age-associated cognitive decline in the large domestic cat population has the potential to contribute substantially to our understanding of neurodegeneration relevant to an aging population of HIV-infected patients.

### 3.2. FIV Neuropathology

**Figure 2:**
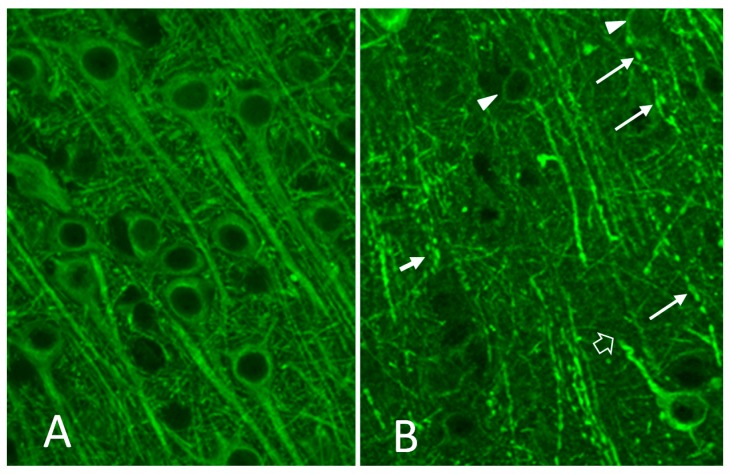
Stain for MAP-2 (green) in the cortex of an FIV-infected cat: (**A**) example of normal cortical pyramidal cells; and (**B**) adjacent areas often show early signs of damage indicated by a fragmented appearance of the MAP-2 stained dendrites and signs of focal swelling.

The CNS pathology that develops in cats experimentally infected with FIV reflects the low grade inflammation seen in pre-AIDS/encephalitis patients and current patients on antiretroviral therapy. Findings include infiltration of perivascular macrophages, microglial activation, astrogliosis, myelin pallor, diffusely distributed signs of neural damage in the form of beaded and/or fragmented dendrites [112,113,114,115,116] (Figure 2A,B). Other studies have shown increased immunoreactivity for neurofilament protein in cortical pyramidal neurons [117] and decreased expression of microtubule associated protein-2 (MAP-2) and glutamic acid decarboxylase (GAD) [118]. In addition, a significant 32% loss of large pyramidal cells within layers two, three and five of the frontal/parietal cortex and large cells within the striatum has been seen in asymptomatic cats approximately three years after infection with a FIV isolated from a naturally infected cat (FIV_NCSU1_) [119]. The loss of neurons correlated with decreases in the CD4:CD8 ratio suggesting a close relationship to systemic immune dysfunction. However, it is worth noting that, although a significant decrease was seen in these specific neuron populations, other neurons were minimally affected (as with HIV) such that the net neuron loss in the cortex was 2.3%. Because the overall loss is modest it is not surprising that these cats typically have deficits that would not be apparent in routine clinical neurologic evaluations. This again parallels HIV infection where sensitive neuropsychological testing is often required to demonstrate underlying cognitive deficits.

**Figure 3:**
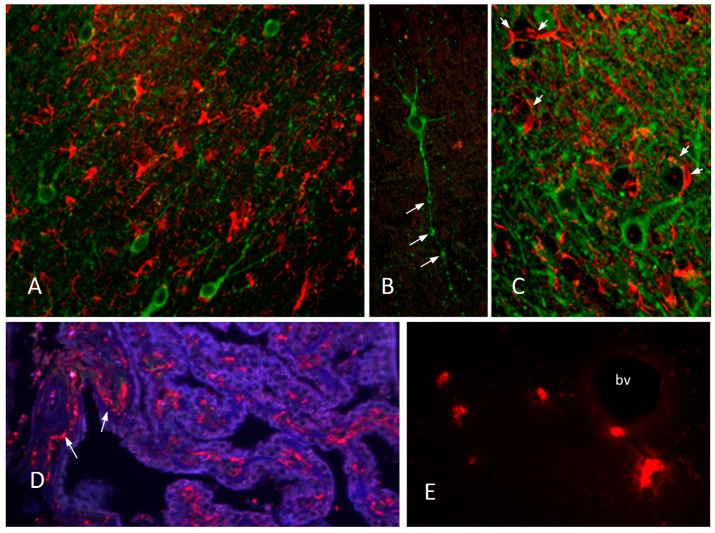
The CNS response to FIV infection. (**A**) Activated microglia stained with an antibody to Iba1 (red) are abundant in the subcortical white matter at the interface between the frontal cortex and basal ganglia. Neurons are stained for MAP-2 (green). (**B**) An example of a single MAP-2+ neuron (green) at the cortical-basal ganglia interface with varicosities in the dendrites indicative of early damage. (**C**) Iba1+ microglia (red) are often seen wrapping neurons in the FIV infected brain but are rare in normal brain. (**D**) An increased number of Iba1+ macrophages (red) populate the choroid plexus in an FIV infected cat. The cuboidal epithelium is stained for cytokeratin (blue). (**E**) Small clusters and scattered macrophages stained for Mac387 (red), a marker of newly migrated macrophages, are seen surrounding blood vessels (bv) in the basal ganglia and cortex (not shown).

With the exception of advanced stages of disease progression where neuron loss becomes evident, the pathology largely reflects ongoing inflammation which is potentially reversible. Figure 3 illustrates inflammatory changes seen in the brain of FIV infected cats at approximately three years post-infection. Iba1 immunoreactive microglia are abundant in white matter (Figure 3A). A subset of neurons stained with MAP-2 show signs of dendritic beading (Figure 3B, arrows) and in subcortical white matter-gray matter boundaries neurons are often seen wrapped by microglia (Figure 3C, MAP-2 stained neurons, green; Iba1 stained microglia, red). Increased numbers of macrophages are seen in the choroid plexus, particularly adjacent to penetrating blood vessels (Figure 3D, Iba1 stained macrophages, red; cytokeratin stained epithelium, blue). Accumulations of Mac387 stained monocyte/macrophages are seen surrounding blood vessels in the basal ganglia (Figure 3E) and cortex. It is notable that, in addition to the inflammatory activity, signs of repair activities can also be seen as increased cortical synaptophysin immunoreactivity in the cortex [119] and increased Timms staining (sprouting) in the hippocampus of FIV-infected cats [120] during the asymptomatic stages of disease. Restorative processes may be mediated by the same cells responsible for inflammatory damage. Studies of human macrophages exposed to HIV have shown that various growth factors and anti-inflammatory cytokines are concurrently secreted with neurotoxic factors indicating a constant balance between toxic and repair processes. Treatments designed to precisely regulate these functions have significant therapeutic potential.

An important observation that deserves greater attention is the development of Tau pathology in aged cats [120,121,122]. Two major efforts in the quest to combat neurodegenerative diseases are focused on understanding the development of Tau pathology and the ability of inflammatory diseases such as HIV infection to enhance age-associated neurodegenerative diseases including Alzheimer’s disease (AD). Experimental models of the disease process are limited since few species other than humans have been shown to develop Tau pathology. The identification of pathological Tau in cats in early studies [121,122] and subsequently in a more detailed study of cats ranging in age from two weeks to 22 years [123] have indicated that aged cats, similar to humans, develop amyloid deposits and pathological changes in Tau. The expression of Tau tangles in the cats was rare indicating that the Tau pathology was still developing or was less severe than that seen in humans with dementia. The appearance of age-related pathology may be at least partially dependent on environmental influences. A study contrasting systemic amyloid deposition in naturally and experimentally infected cats identified deposits in 35% of the naturally infected cats [124] but no amyloid accumulation in experimentally infected specific pathogen free cats. This observation is an important reminder that factors such as immune activation/inflammation are likely to play a significant role in the development of pathology. It is recognized that a large percentage of domestic cats will develop feline cognitive dysfunction as they reach ages >10 years [125,126]. Identification of the early processes leading to age-dependent Tau pathology is critical to our understanding of the natural disease process and studies of cat aging may be a significant source of new insights.

## 4. Mechanisms of FIV Neurotoxicity

### 4.1. Studies Using Cultured Fetal Feline Cells

**Figure 4:**
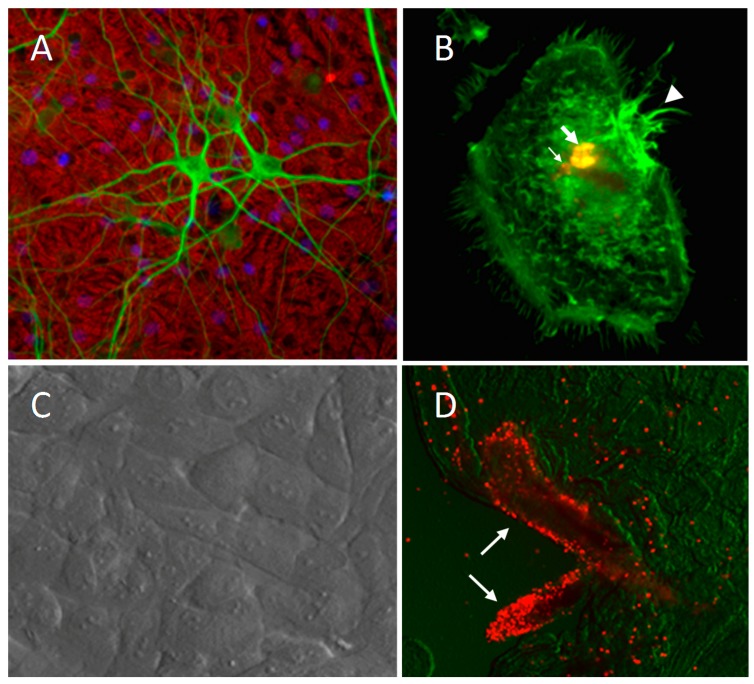
Examples of feline cells grown in culture for basic research on neuro-immune interactions and FIV neuropathogenesis. Excellent recognition of feline proteins is seen with a variety of antibodies to standard markers. (**A**) Neurons identified with MAP-2 immunocytochemistry (green) on a bed of GFAP immunoreactive astrocytes (red). (**B**) A cultured feline choroid plexus macrophage stained with an antibody to FIV (red) and counterstained with phalloidin (green) to show actin structure. FIV is compartmentalized within the cells (large arrow) and can be also seen in small endosome-like structures (small arrow). Ruffles are expressed on the surface of the cell and prominently on one side (arrowhead). (**C**) Cultured feline microvascular endothelial cells viewed with differential interference contrast. (**D**) Macrophages stained with DiI-acetylated-LDL (red) are prominent surrounding penetrating vessels in a whole mount preparation of choroid plexus illustrating the abundance of these cells at the blood-CSF interface.

The availability of fetal tissue from cats provides the opportunity to perform in vitro experiments that explore the cellular processes that lead to pathology. Neurons are easily cultured from fetuses at approximately 35–40 days gestation (~6–7 cm fetus) [21,111,127,128,129,130]. In addition, microglia, choroid plexus epithelium and choroid plexus macrophages have been cultured from the fetal brain [77,90,131] and microvascular endothelial cells have been cultured from late gestation brain tissue [83,132,133]. Figure 4 illustrates various cultures prepared from fetal cat brain. Studies of FIV interactions with these cells have led to a number of important insights into the cellular processes associated with calcium dysregulation and neural dysfunction.

In early studies of virus interactions with primary feline mixed neuronal cultures (e.g., Figure 4A), the addition of FIV produced minimal cell death but, instead, primed the neurons for damage in response to a normally subtoxic excitatory challenge with glutamate [129]. The concentration-effect curve for glutamate toxicity was shifted approximately three-fold to the left indicating an increase in the neuronal sensitivity to the glutamate. The effect of FIV developed gradually for up to seven days suggesting a cumulative release of soluble toxic factors, presumably from microglia. Subsequent studies using choroid plexus macrophages (Figure 4B) demonstrated that FIV induced the release of potent “toxins” that caused a dysregulation of calcium homeostasis in the neurons [127]. Although small amounts of FIV provirus were detected in some of these cultures at 17–24 days post-inoculation, no evidence of productive infection was observed during the period of toxin generation that peaked between approximately 3–6 days. A comparison of live virus, inactivated virus and viral envelope protein showed that each could induce the same secretion of neurotoxic factors from macrophages demonstrating that the inflammatory response leading to neuronal damage was independent of infection [128]. Other studies described above have used preparations of endothelial cells (Figure 4C) and choroid plexus (Figure 4D) for trafficking studies.

### 4.2. FIV and Intracellular Calcium Homeostasis

**Figure 5:**
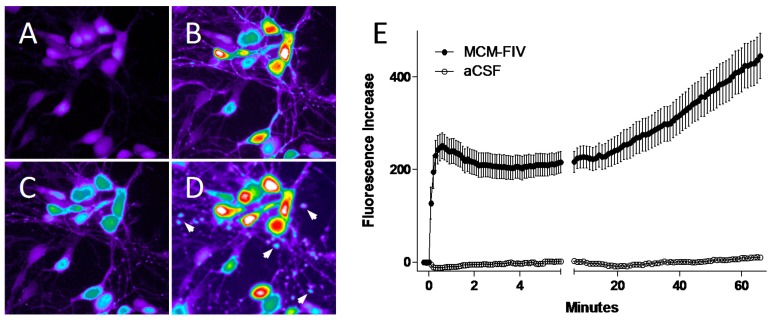
Exposure of cultured feline cortical neurons to soluble factors secreted by choroid plexus macrophages infected with FIV results in a dysregulation of calcium homeostasis followed by cytoskeletal damage. (**A**) Pseudocolored image showing resting calcium in feline cortical neurons. (**B**) Acute increase in calcium triggered by addition of conditioned medium from macrophages inoculated with FIV. (**C**) Partial recovery of calcium is typically seen over the first few minutes. (**D**) The acute increase is followed by a gradual, progressive increase in intracellular calcium. This delayed deregulation of calcium triggers the development of focal swellings in dendrites and axons (beading), a reversible hallmark of early pathogenesis. Similar results are seen with inactivated virions or FIV surface glycoprotein (envelope) indicating that infection is not necessary to provoke the secretion of neurotoxic factors. (**E**) Example of the average profile of calcium increase in response to macrophage conditioned medium (MCM) relative to normal basal calcium levels in cells treated with artificial cerebrospinal fluid (aCSF). Beading typical appears at approximately 20–30 min during the delayed rise and is independent of the acute increase.

The identity of the soluble factors that impair neuronal function is still largely unknown. While a diverse range of HIV-associated, macrophage-derived factors have been proposed to be responsible for neurotoxicity [4,134,135,136,137], none have been shown to recapitulate the effects of the macrophage supernatant on neural calcium homeostasis and damage (which mimics pathology in vivo). Most investigators agree that accumulation of intracellular calcium is a pivotal neural response early in the pathological cascade. Thus, stabilization of intracellular calcium has been a focus of many efforts to prevent damage [138,139]. Studies with primary feline neurons showed that the accumulation of intracellular calcium was due to a gradual delayed increase that could be dissociated from acute increases in calcium [127,130]. An example of a typical response of feline neurons to conditioned medium from choroid plexus macrophages is shown in Figure 5.

When conditioned medium from FIV-treated choroid plexus macrophages was added to cultured feline neurons (1:5 dilution) an acute increase in intracellular calcium was seen (Figure 5B) followed by partial recovery (Figure 5C) and then a gradual delayed rise to high levels with structural damage in the form of beading (Figure 5D, arrows). The typical average calcium response in Figure 5E illustrates the acute rise, sustained plateau and the gradual delayed rise necessary for the development of cytoskeletal damage. Basal calcium in control cultures does not change over the same time course. The conditioned medium always provoked a much greater toxic response than cell-free FIV virions, consistent with the idea that macrophage activation is a primary cause of neurotoxicity. These early pathological changes are reversible but, if the exposure is sustained, the beading is followed by dendritic pruning and eventually cell death. The reason for the delayed rise is not completely understood. However, studies with HIV-infected human CSF have indicated that a loss of sodium-calcium exchanger (NCX) function contributes to the calcium destabilization [140] by reducing the ability to export excess intracellular calcium. This deficit would synergistically increase the magnitude of the intracellular calcium rise regardless of the source of calcium entry into the cytosol [127]. Evidence from these studies indicate that NMDA glutamate receptor activity makes the greatest contribution to pathology, consistent with numerous studies using different models of HIV neuropathogenesis [137,141,142,143,144,145,146]. Another mechanism hypothesized to lead to enhancement of glutamatergic activity is the inhibition of astrocyte glutamate transporters [147] resulting in prolonged exposure of neurons to glutamate. FIV infection of astrocytes in vitro resulted in a 50%–60% reduction in the ability to transport glutamate [76]. The extent of this effect in vivo is still unclear but increased concentrations of glutamate have been reported in extracts prepared from brains of FIV-infected cats [71].

Overall, these studies illustrate that inflammation sensitizes neurons in a fashion that encourages the development of more severe neurodegenerative pathologies. Therapeutic approaches that prevent the early neuronal dysfunction have the potential to interrupt the pathological cascade at a point where it is largely reversible. The FIV model offers many benefits for these efforts. Nevertheless, it is also important to recognize limitations of the model. Lack of species-specific reagents and assays can prevent detailed exploration of toxic factors and mechanisms. In spite of these limitations, the FIV model offers the ability to assess potential neuroprotective strategies both in vitro and in vivo.

## 5. Development of Neuroprotective Treatments

**Figure 6:**
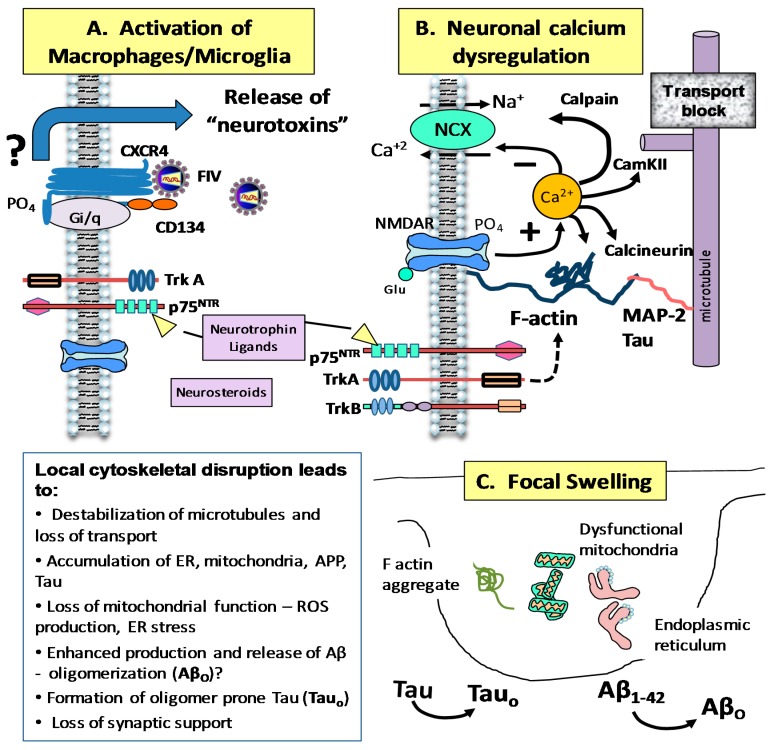
Summary of inflammation-associated neuropathogenesis in response to FIV. (**A**) FIV infects monocytes and macrophages through interactions with CD134 and CXCR4. The virus rapidly gains access to the brain via transmigration of infected monocytes at the blood brain barrier or macrophages at the blood-CSF barrier and establishes what is thought to be a self-sustaining reservoir within microglia. Activation by FIV results in the release of factors that have toxic effects on neurons. The processes that control toxin release are not well understood but appear to be regulated in part by neurotrophins. (**B**) Factors (“Neurotoxins”) in the secretome of the mononuclear phagocytes enhance neuronal calcium signaling in response to glutamate and inhibit the ability of neurons to recover to resting calcium levels. The loss of recovery is thought to be due to a loss of sodium-calcium exchanger (NCX) function. The net result is a gradual, sustained accumulation of calcium that disrupts the cytoskeleton. Activation of calcium-dependent enzymes such as calpain, CamKII, and calcineurin may contribute to actin destabilization and loss of structure. (**C**) The loss of structure disrupts transport and results in focal swelling (beading) of axons and dendrites. The accumulation of mitochondria, endoplasmic reticulum, amyloid precursor protein (APP) and Tau in the swellings may set the stage for oxidative stress and potential cleavage of oligomerization prone Abeta and Tau. The p75 neurotrophin receptor ligand LM11A-31 reduces calcium accumulation and protects the cytoskeleton. Neurosteroids may offer protection by reducing inflammation.

Insights from the above studies have supported a model of neuropathogenesis illustrated in Figure 6. FIV penetration into the nervous systems via infected monocytes establishes a viral reservoir with chronic activation of macrophages and microglia. The subsequent inflammatory response, triggered in part by putative FIV interactions with CXCR4, causes the release of soluble factors that act on neurons to provoke a dysregulation of calcium homeostasis. Progressive accumulation of calcium due to decreased export of calcium in conjunction with NMDA receptor activity drives pathological changes in the cytoskeleton through mechanisms that are not well understood but may include calcium-dependent activation of calpain, calcium-calmodulin protein kinase II (CamKII) or calcineurin. The net result is the loss of actin structure, inefficient transport and the development of focal swellings in the dendrites and axons (beading) where organelles, amyloid precursor protein and Tau accumulate.

Neurotrophin ligands may act at receptors on neurons or macrophages/microglia to prevent the calcium dysregulation and cytoskeletal disruption. Because FIV neuropathology and neurodegenerative mechanisms are highly similar to HIV it provides the opportunity to test potential therapies in FIV infected cats. Studies of cultured feline neurons have shown that a novel neurotrophin ligand which targets the p75 neurotrophin receptor (p75^NTR^) has strong neuroprotective efficacy. At 10 nM, the ligand protected neurons from the long-term toxic effects of FIV in mixed neural cultures, suppressed the delayed accumulation of intracellular calcium (but not the acute increase) and greatly decreased dendritic damage [130]. The in vivo efficacy of the compound is currently being assessed in FIV infected cats.

In a separate series of studies Maingat et al. [53] demonstrated the potential for neurosteroids to prevent FIV associated degeneration. Treatment of experimentally infected cats with sulfated dehydroepiandrosterone (DHEA-S) reduced inflammation and prevented behavioral deficits and neuron loss indicating that neurosteroids could be useful for the treatment of inflammation-mediated neurodegeneration. Together, these studies [53,130] show that the FIV model parallels the development of neurodegenerative disease in humans and can be used to identify therapeutic strategies that protect the nervous system.

## 6. Conclusions

FIV infection of the nervous system shares many common features with HIV infection including usage of the co-receptor, CXCR4, similar cellular targets, rapid penetration and infection of the CNS, the generation of a diffuse CNS inflammatory response and gradual, progressive pathogenesis. Although there are differences between FIV and HIV virus structure, primary receptor usage and severity of CNS disease, the basic mechanisms that disable neurons appear to be almost identical providing the opportunity to investigate disease mechanisms. In vitro and in vivo studies of FIV have provided insights into: (1) the natural progression of CNS disease; (2) the role of astrocytes and microglia in immune cell trafficking across the blood-brain and blood-CSF barriers; (3) the mechanisms of early infection, viral diversification and disease progression in the CNS; (4) the potential importance of the choroid plexus blood-CSF barrier as a site of virus entry; (5) the potential mechanisms that lead to the loss of neuronal calcium homeostasis and neuronal pathology; and (6) have facilitated the identification and testing of novel treatments. The FIV model recapitulates many features of inflammation-associated neurodegeneration that are thought to promote various neurodegenerative diseases. The ability to investigate the pathogenesis of FIV in specific-pathogen free cats in parallel with in vitro studies of cultured feline cells provides the opportunity for translational studies that should facilitate the design and evaluation of new therapeutic strategies that prevent neurodegeneration.
